# A Rare Case of Henoch-Schönlein Purpura and Mycobacterium xenopi Pulmonary Infection

**DOI:** 10.7759/cureus.13533

**Published:** 2021-02-24

**Authors:** Jad Saliba, Maximilien Grall, Christian Saliba

**Affiliations:** 1 Internal Medicine, Centre Hospitalier Universitaire Charles Nicolle Hospital, Rouen, FRA; 2 General Surgery, Lebanese American University-Medical Center, Beirut, LBN

**Keywords:** henoch-schönlein purpura, mycobacterium xenopi, leukocytoclastic vasculitis, mycobacteria

## Abstract

Henoch-Schönlein purpura (HSP) is a small-vessel vasculitis with cutaneous, articular, gastrointestinal, and renal manifestations. Leukocytoclastic vasculitis and IgA deposits are classically found when involved skin and kidneys are biopsied. The disease's etiology remains unknown, although many bacterial and viral infections have been described as triggering factors.

A 53-year-old woman presented with fever, arthralgia, and non-thrombocytopenic purpura. She also had a segmental pulmonary collection with peripheral alveolar consolidation. *Staphylococcus aureus* and mycobacteria growth was found on sputum cultures. In addition to intravenous antibiotics and anti-mycotic drugs, high-dose corticosteroids were urgently administered due to the development of severe intestinal symptoms. A cutaneous biopsy later confirmed HSP. Microbial identification yielded *Mycobacterium xenopi*.

In the review of the literature, we only found 12 cases of *Mycobacterium tuberculosis* and one case of *Mycobacterium avium-intracellulare* complex that were associated with HSP. Nearly, half of the cases responded to anti-mycotic treatment alone. The rest required immunosuppressants.

We report the first case of *M. xenopi* pulmonary infection in HSP. This disease process can have a severe course, which requires rapid recognition and treatment.

## Introduction

Henoch-Schönlein purpura (HSP) or immunoglobulin A vasculitis (IgAV) is a systemic small-vessel vasculitis. It usually presents as an acute onset of palpable purpura or petechiae, associated with systemic symptoms involving the gastrointestinal tract, musculoskeletal system, and/or the kidneys.

Histologically, leukocytoclastic vasculitis is a characteristic skin biopsy finding. Direct immunofluorescence usually reveals IgA and complement deposition in blood vessels of the upper dermis. IgA mesangial deposits are typically found when the kidneys are involved. In some cases, we can also see extracapillary proliferation.

There is a seasonal variation in HSP’s incidence, with high rates in the fall, winter, and spring. It rarely occurs during summer. Two-thirds of patients with this disease are previously affected by acute upper respiratory illnesses. This raises the suspicion of a possible connection between IgAV (HSP) and infections [1].

## Case presentation

A 53-year-old woman presented with four days of ongoing fever, accompanied by progressive bilateral pain over the calves, that rapidly extended to the knees, ankles, and wrists. Cutaneous eruption over the lower extremities followed her articular symptoms. The patient also complained of fatigue and had been admitted to a weight loss of 5 kg over two weeks. She was treated two weeks prior for a pulmonary infection with Cefpodoxime for seven days. No pulmonary, urinary, or abdominal symptoms were noted on admission.

The patient is a current smoker (50 pack/year) with is a long history of excessive alcohol consumption (>3 glasses of wine/day). She suffered from pulmonary emphysema and recurrent pneumothorax which required pleurodesis in 2001. She also had a history of positive *Aspergillus fumigatus* pulmonary sputum culture.

On physical examination, both ankles were erythematous and swollen. Palpable purpuric lesions were covering both lower limbs and the abdomen. Heart and lung auscultation were normal; the abdomen was soft and non-tender.

Blood tests on admission showed an elevated C reactive protein (200 mg/dl) and ferritin (344 mmol/l) levels and low albumin (23 g/l). The rest of the analysis was normal and included a complete blood count, hepatic and renal chemistry profiles, urinalysis, thyroid-stimulating hormone, complement level, protein electrophoresis as well as a complete auto-immune panel with anti-neutrophil cytoplasmic antibodies and cryoglobulin.

**Figure 1:**
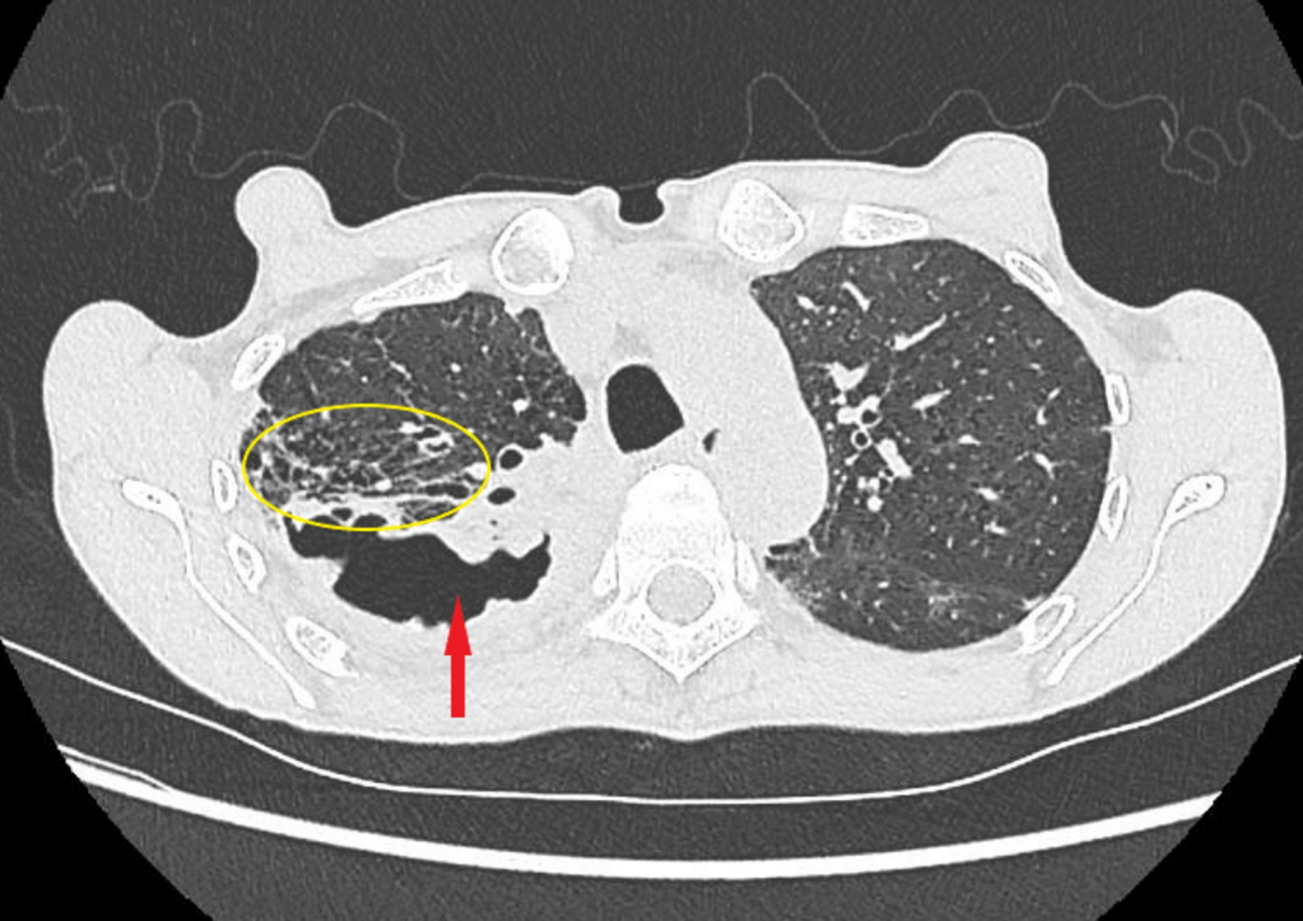
Image showing thick-walled right-sided segmentum apicale cavitation (red arrow) with peripheral alveolar consolidation and infiltrates (yellow circle).

Thoracic computed tomography (CT) scan demonstrated thick-walled right-sided segmented apical lesion with peripheral alveolar consolidation and right mediastinal and hilar adenomegaly (Figure 1). Post-bronchoscopy sputum examination was acid-fast bacilli smear-positive and culture was positive for *Staphylococcus aureus*.

The patient was first treated with Colchicin 1 mg that helped reduce pain and articular swelling. Few days after admission, she suddenly complained of severe diffuse abdominal pain, vomiting and her physical examination was remarkable for abdominal guarding and hypotension. Urgent abdominal CT scan showed signs of significant duodenal inflammation. Purulent cough with hemoptysis were other remarkable clinical symptoms that appeared later during hospitalization.

Despite the fact that the diagnosis was unclear, the clinical evolution was consistent with the severe intestinal pathology of systemic disease. The decision was made to administer an intravenous infusion of Methylprednisolone 1 mg/kg/day. The patient was also receiving Piperacilline/Tazobactam to cover intra-abdominal organisms, Oxacillin for pulmonary infection, and wide-spectrum anti-mycotic agents (Isoniazid, Rifabutin, Pyrazinamide, and Clarithromycin).

Skin biopsy showed leukocytoclastic vasculitis, consistent with HSP, with immunoglobulin IgA staining on immunofluorescence. Pulmonary aspiration smear was acid-fast bacilli positive and sputum culture yielded *Mycobacterium xenopi*.

**Figure 2:**
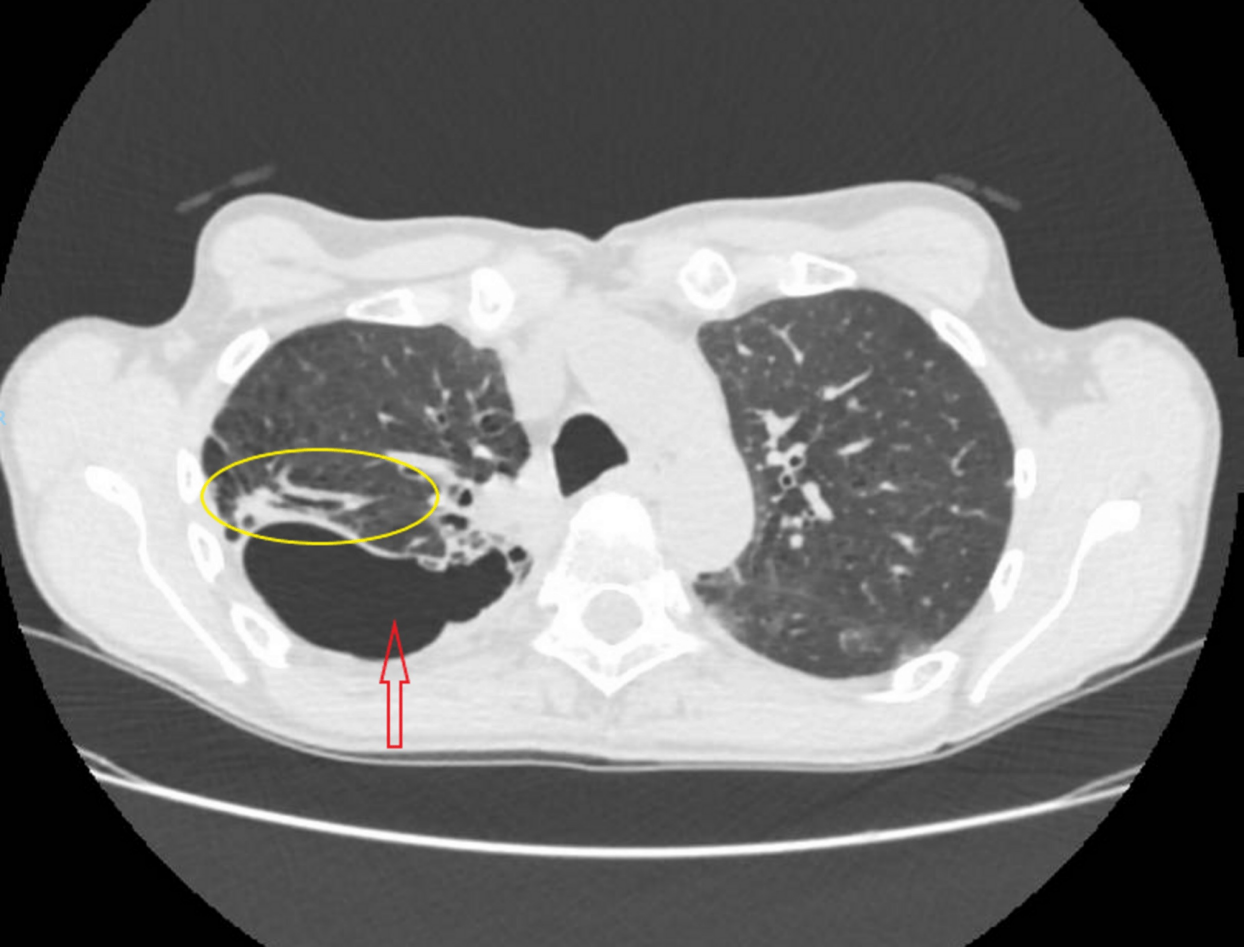
Image showing the important regression of the alveolar infiltrates (yellow circle) with mild regression of the apical cavitation (red arrow) six months following treatment.

The patient responded to treatment. Pulmonary lesions decreased on control imaging at three and six months (Figure 2). No recurrence of symptoms was noted, which permitted a progressive reduction of corticosteroids. No renal disease was found on regular screening during follow-up.

## Discussion

To the best of our knowledge, this is the first case that describes an association between *M. xenopi* infection and HSP. *M. xenopi* is a slow-growing non-tuberculous mycobacterium (NTM) that contaminates water sources and instruments (e.g., bronchoscopes). It has the ability to survive at 36-50 °C temperatures; hence, it is found in hot water systems. Infection can occur after water ingestion or aerosol inhalation (e.g., showers).

Pulmonary infection is the most common clinical manifestation. *M. xenopi* infection has a predilection for structural respiratory diseases; chronic obstructive pulmonary disease (COPD) and emphysema being the most common predisposing factors. Prevalence is highest in Europe and Canada [2]. Cavitations are common radiological findings, usually of >2 cm in size. Good clinical response and radiological improvement are obtained with either rifampicin plus ethambutol or rifampicin, ethambutol, and isoniazid regimen.

Our patient presented with general symptoms, purpura, and atypical radiologic findings suggestive of pulmonary infection and sputum culture growing for *Staphylococcus aureus* and mycobacteria. Severe intestinal involvement pointed towards the diagnosis of small vessel vasculitis and required urgent immunosuppressive therapy despite infection. Leukocytoclastic vasculitis on skin biopsy with immunoglobulin IgA staining on immunofluorescence later confirmed HSP. *M. xenopi* identification in sputum culture was an unexpected finding as it revealed a rare association between atypical mycobacterial infection and HSP, which resolved with anti-mycotic treatment and corticosteroids.

In the review of the literature, we found no similar case of *M. xenopi* related to HSP. The closest report was a non-tuberculous mycobacterial infection in a patient who later developed HSP. This case was described by Yano in 2004 who illustrated a *Mycobacterium avium-intracellulare* complex pulmonary infection followed by HSP clinical manifestations that resolved after prednisolone administration [3]. On the other hand, many reports showed *M. tuberculosis* (TB) infection as a predisposing factor in HSP. These cases are summarized in Table 1. All patients improved either with anti-tuberculosis drugs (ATD) alone or with ATD associated with CS [4-15].

**Table 1:** The case reports of Henoch-Schönlein purpura associated with tuberculosis. ATD: anti-tuberculosis drugs, CS: corticosteroids, EB: ethambutol, INH: isoniazid, PZA: pyrazinamide, RFP: rifampicin, SM: streptomycin, TB: tuberculosis.

Case (year)	Age (years)/sex	Tuberculosis sites	HSP timing	Histology	Treatment
Pacheco [4]	33/male	Pulmonary TB + lymphadenitis	After TB, before ATD	Skin biopsy: deposition in the walls of the small vessels of IgA. IgM, C3, and C1Q	ATD + CS
Washio et al. [5]	21/male	Tuberculous pleuritis	Two weeks after TB symptoms, before ATD therapy	Renal biopsy: a focal segmental increase in the mesangial matrix, granular deposits of IgA and C3 in the mesangial areas	INH + RFP + EB + CS
Saatci et al. [6]	7/female	Urinary tract TB	Before TB symptoms	Skin biopsy: leucocytoclastic vasculitis	ATD
Mishima et al. [7]	34/male	Pulmonary TB	5 months after TB treatment	Not performed	INH + RFP + EB + CS
Han et al. [8]	41/male	Disseminated TB	15 days after TB symptoms and treatment	Skin biopsy: leucocytoclastic vasculitis. Renal biopsy: mesangial IgA, IgG, and C3 deposits.	INH + RFP + EB + PZA
Islek et al. [9]	8/female	Pulmonary TB	Concomitant with TB	Skin biopsy: leucocytoclastic vasculitis and IgA deposition	INH + RFP + PZA
Kuboi and Nomura [10]	12/female	Renal TB	1 month after TB symptoms and treatment	Skin biopsy: leucocytoclastic vasculitis	INH + RFP + EB + PZA
Chemli et al. [11]	46/male	Tuberculous pleuritis	After TB symptoms and treatment	Skin biopsy: anaphylactoid purpura	ATD + topical steroids
Kitamura et al. [12]	38/male	Pulmonary TB	3 months after TB treatment	Skin biopsy: leucocytoclastic vasculitis with fibrinoid necrosis, IgA, C3, and C4 deposition. Renal biopsy: mesangial IgA and C3 deposits.	INH + RFP + EB + PZA + CS
Isobe et al. [13]	54/male	Pulmonary TB	Concomitant with TB	Skin biopsy: leukocytoclastic vasculitis	INH + RFP + EB + PZA + CS
Alvarez et al. [14]	6/male	Pulmonary TB	Concomitant with TB	Not performed	ATD
Muchahary et al. [15]	60/male	Disseminated TB	Concomitant with TB	Renal biopsy: a mild increase in mesangial matrix and cellularity with focal endocapillary proliferation in two glomeruli, partial cellular crescent in one glomerulus, diffuse granular deposition of IgA in the peripheral capillary walls and mesangium.	INH + SM + RFP + EB + PZA

**Figure 3:**
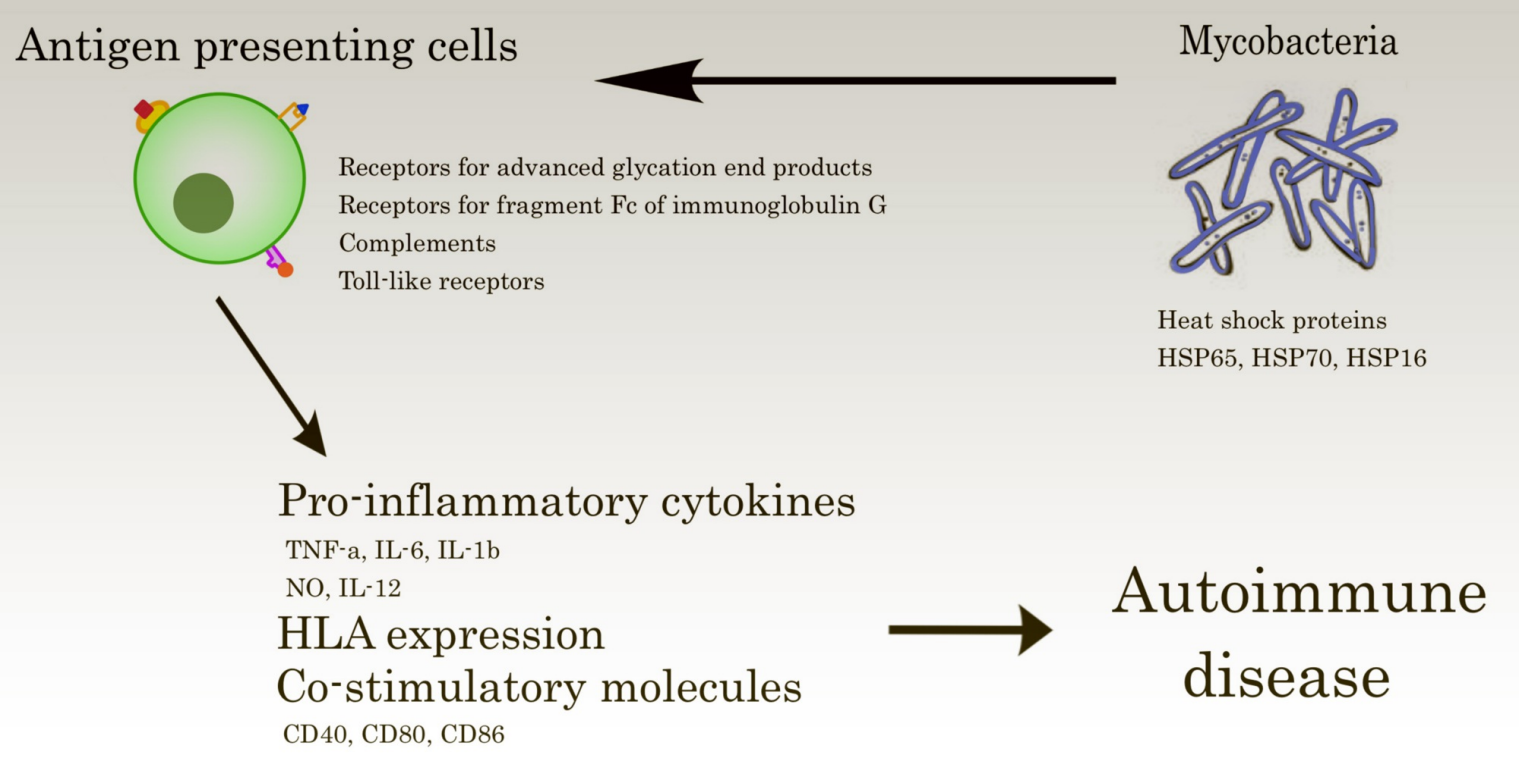
Image showing microbial heat shock protein pathogen-associated molecular patterns that are recognized by pattern recognition receptors on/in antigen-presenting cells and may induce autoimmunity in genetically predisposed hosts.

The cause of HSP remains unclear despite all these described associations. Laboratory studies that focused on the pathophysiology of this intriguing correlation revealed possible theories. Mycobacteria can trigger auto-immune diseases in predisposed hosts when microbial heat shock protein pathogen-associated molecular patterns (PAMPs) are identified by pattern recognition receptors. This molecular recognition induces cellular signaling, which stimulates pro-inflammatory cytokine production [16]. It also promotes human leukocyte antigens (HLAs) and co-stimulatory molecules (CD40, CD80, and CD86) expression. These modifications lead to T helper (Th)1/Th2 mediated immune response and/or autoimmunity (Figure 3) [2].

Other researchers have shown that mycobacteria also inhibit apoptosis of infected macrophages by regulating certain types of heat shock proteins [17] and by prompting serine protease inhibitors [18,19]. There is also a lack of apoptotic cell clearance during mycobacterial infections [20]. Both mechanisms explain the relationship between mycobacterial infection and autoimmunity.

Antigen-presenting cells (APC) can express mycobacterial antigens on their surface. CD4+ and CD8+ T cells recognize these antigens and activate B lymphocytes using HLA class I and II molecules. This interaction can generate an aberrant autoimmune response. Mycobacterial antigens can also induce autoimmune reactions by slowing APC bone marrow maturation, which dysregulates Th1/Th2 profiles, disturbs T-cell tolerance, and alters co-stimulatory molecules and regulatory cells’ expression [2].

## Conclusions

In this article, we describe the first case of *M. xenopi* infection triggering HSP. The association between atypical mycobacterial infections and HSP is rare and the true mechanism still remains to be clarified. This clinical scenario highlights the severity of this condition and the importance of early suspicion and prompt management.
